# A Novel Approach to Assessing Infant and Child Mental Rotation

**DOI:** 10.3390/jintelligence11080168

**Published:** 2023-08-20

**Authors:** Aaron G. Beckner, Mary Katz, David N. Tompkins, Annika T. Voss, Deaven Winebrake, Vanessa LoBue, Lisa M. Oakes, Marianella Casasola

**Affiliations:** 1 College of Human Ecology, Cornell University, Ithaca, NY 14853, USA; 2 Department of Psychology and Center for Mind and Brain, University of California, Davis, CA 95618, USA; 3 Department of Psychology, Rutgers University, Newark, NJ 08854, USA

**Keywords:** mental rotation, eye tracking, spatial thinking, infants, young children, staircase procedure

## Abstract

Mental rotation is a critically important, early developing spatial skill that is related to other spatial cognitive abilities. Understanding the early development of this skill, however, requires a developmentally appropriate assessment that can be used with infants, toddlers, and young children. We present here a new eye-tracking task that uses a staircase procedure to assess mental rotation in 12-, 24-, and 36-month-old children (N = 41). To ensure that all children understood the task, the session began with training and practice, in which the children learned to fixate which of two houses a giraffe, facing either left or right, would approach. The adaptive two-up, one-down staircase procedure assessed the children’s ability to fixate the correct house when the giraffe was rotated in 30° (up) or 15° (down) increments. The procedure was successful, with most children showing evidence of mental rotation. In addition, the children were less likely to succeed as the angle of rotation increased, and the older children succeeded at higher angles of rotation than the younger children, replicating previous findings with other procedures. The present study contributes a new paradigm that can assess the development of mental rotation in young children and holds promise for yielding insights into individual differences in mental rotation.

## 1. Introduction

Mental rotation is a critically important spatial cognitive skill that appears to emerge in infancy (e.g., Moore and Johnson 2008) and undergoes development across childhood (e.g., Estes 1998; Frick et al. 2013; Pedrett et al. 2023). Moreover, mental rotation is related to the development of other spatial skills (Mix et al. 2016; Newcombe et al. 2019) and is associated with mathematical achievement (Cheng and Mix 2014; Frick 2019; Mix et al. 2016; Verdine et al. 2014) and entry into the Science, Technology, Engineering, and Mathematical (STEM) fields (Newcombe and Frick 2010; Shea et al. 2001; Wai et al. 2009). This association, paired with gains in mental rotation and other spatial skills following training (Uttal et al. 2013; Xu et al. 2023), has motivated recommendations to incorporate spatial activities into classrooms and develop educational resources for parents to bolster the early development of these skills. Indexing mental rotation is a critical component to outlining its development and assessing the impact of experience on the development of spatial skills.

Tools are needed for understanding the development of mental rotation across early childhood. Despite a large amount of research on mental rotation in infancy (Moore and Johnson 2020) and childhood (Möhring et al. 2021), we do not yet have a coherent understanding of the development of mental rotation across this age range. Studies have revealed evidence of mental rotation in the first year after birth (Christodoulou et al. 2016; Erdmann et al. 2018; Frick and Möhring 2013; Hespos and Rochat 1996; Lauer et al. 2015; Möhring and Frick 2013; Moore and Johnson 2008, 2011; Quinn and Liben 2008, 2014; Slone et al. 2018), whereas other studies have shown that even children of three to five years do not display evidence of mental rotation in some tasks (e.g., Estes 1998; Frick et al. 2013; Pedrett et al. 2023). This discrepancy for when mental rotation is first seen may arise from the use of different tasks for infants versus young children. Specifically, mental rotation has been assessed in different age groups using tasks that are tailored to their developmental level, which means that tasks used with younger and older children vary in their reliance on verbal vs. non-verbal responses, the use of rewards, and memory demands, among other things. Although such variations are necessary when developing tasks for children of different ages, the use of tasks that vary in these ways creates challenges for tracing the development of mental rotation. Thus, the lack of a single procedure for indexing mental rotation across a wide age range in the first years of life represents a barrier to addressing gaps in the literature and appreciating mental rotation as a cognitive skill that matures across a protracted developmental period. The goal of this investigation was to develop a single assessment of mental rotation that is developmentally appropriate from infancy through the preschool years.

Studies of mental rotation in infancy have used a variety of looking-time procedures, such as habituation (Moore and Johnson 2008; Slone et al. 2018), change detection (Lauer et al. 2015), familiarization (Quinn and Liben 2014), and violation of expectation (Möhring and Frick 2013). As is typical in studies of cognition in infants, these tasks rely on an indirect measure of infants’ mental rotation ability. Specifically, these tasks leverage infants’ interest in novelty and change. For example, in habituation and familiarization, infants first see an image or object in one or more orientations, and then their looking at that object in a new orientation vs. a mirror image of that object is compared (Christodoulou et al. 2016; Constantinescu et al. 2018; Erdmann et al. 2018; Hespos and Rochat 1996; Moore and Johnson 2008, 2011; Quinn and Liben 2008, 2014; Slone et al. 2018). In violation of expectation, the object is typically moved behind an occluder over a period of many seconds (apparently positioning the object in a new orientation) and the occluder is later removed to reveal a non-mirrored (possible) or mirrored (impossible) version of the original object. Mental rotation is inferred from infants’ visual preference for mirrored objects compared to the non-mirrored object (Möhring and Frick 2013). In change detection tasks, infants view stimulus streams in which objects briefly appear and disappear, each time in a new orientation (Lauer et al. 2015). Infants’ mental rotation is inferred from their preference for streams in which a mirror-image object appears compared to streams in which the same object reappears in new orientations. In these tasks, infants’ performance is typically assessed on one to four trials, and thus infants are tested on very few different orientations. In addition, the tasks used with infants vary considerably in whether infants must use long-term memory representations to recognize mental rotations, whether infants must mentally rotate using visual short-term memory, and how easily infants can compare novel rotations to mirror images of familiar objects (Beckner et al. 2023). These design features likely impact observed findings in existing infant mental rotation studies.

In contrast to the procedures used in infant studies, studies of older children typically administer tasks similar to those used with adults. These tasks require explicit instructions and measure overt behavioral responses. For example, studies with older children may use choice tasks in which children are provided with multiple images, often identical images at different angles of rotation, and they are asked to identify which items are rotated versions of a target and which are mirror images of a target. These tasks allow researchers to present participants with multiple trials, examine explicit measures of mental rotation, and critically examine how angle of rotation impacts participant performance within-subject. Nevertheless, implementing these kinds of tasks to index mental rotation across infancy and early childhood presents challenges. The first challenge is that mental rotation tasks that involve choice, explicit instructions, and index mental rotation from overt behavioral responses are not well suited for prelinguistic infants. Studies using such tasks have often failed to reveal above-chance performance in children under 4 years of age (Levine et al. 1999). That is, the features of child mental rotation tasks that make them like adult mental rotation procedures are the same characteristics that present challenges for studying prelinguistic infants and toddlers.

The second challenge is that there are a wide range of tasks for indexing mental rotation in children, and tasks designed for children of the same age group can vary considerably. Studies with preschoolers and young school-aged children use tasks such as the Picture Rotation Task (Quaiser-Pohl 2003), Children’s Mental Transformation Task (Levine et al. 1999), Brick Building Task (Aguilar Ramirez et al. 2021), the primary mental abilities–space relations test (Thurstone 1957), or other tasks (Estes 1998). These tasks often index different measures of success to indicate mental rotation (e.g., manually placing a piece into a puzzle vs. building a configuration vs. selecting a response option vs. pointing) and vary in terms of the complexity of stimuli (e.g., animals vs. abstract shapes), the number of selection options available, the motor demands (e.g., button press vs. point vs. object manipulation vs. verbal response vs. circling an answer on a paper form), and the method of administration (e.g., paper forms vs. Zoom vs. touchscreens vs. computers vs. naturalistic play). Just as procedural variations are a potential explanation for disagreements in the infant mental rotation literature (Beckner et al. 2023; Moore and Johnson 2020, such differences may also drive inconsistent findings regarding the factors that impact mental rotation in children.

There are at least two approaches that may help resolve these methodological challenges: (1) administering existing infant mental rotation procedures to older participants with the goal of determining their appropriateness for these children or (2) developing a novel task for indexing mental rotation across a wider age range from infancy to early childhood. Mental rotation tasks that measure looking time are a promising methodological tool for bridging the conceptual gaps in our understanding of mental rotation from infancy through early childhood. In fact, studies have adapted a violation-of-expectation procedure to assess mental rotation in toddlers (Pedrett et al. 2020) and preschool-aged children (Pedrett et al. 2023). In a series of studies conducted by Pedrett et al., children were initially familiarized with a rotating object that disappeared behind an occluder during the later phases of familiarization. After familiarization, children were shown four test arrays containing mirror and non-mirror versions of the rotating shape. As is typical in infant studies using this procedure, children’s mental rotation was inferred from their looking at the mirror and non-mirror objects presented in the test arrays. However, in contrast to infants tested in similar violation-of-expectation tasks (Möhring and Frick 2013), neither the toddlers nor the preschoolers exhibited longer looking at the mirrored image. These results suggest that conventional measures of infant mental rotation failed to reveal evidence of mental rotation in toddlers (Pedrett et al. 2020) or preschool-aged children (Pedrett et al. 2023). It is noteworthy that other aspects of their findings—such as children’s anticipatory looking to the location where the rotating shape would appear immediately after occlusion—were indicative of mental rotation, but the authors concluded that children’s anticipatory looking in this specific procedure reflected a less sophisticated cognitive process than what is typically measured in mental rotation studies. That is, aspects of children’s looking behavior indexed mental rotation in the VOE procedure, but not in the same way that is typically reported in infant studies.

Although a promising first step, the work by Pedrett et al. (2020, 2023) highlights the challenges that arise when infant looking-time procedures are used to index mental rotation in older children. Not only did older children fail to show evidence of mental transformation of objects in a violation-of-expectation procedure, but this task cannot be used to systematically examine angular disparity effects because it involves a limited number of trials^1^. In the present study, we adopt a different approach, developing a novel eye-tracking task to assess mental rotation in children ranging in age from 12 months to 36 months. Several aspects of our task address the challenges in bridging mental rotation findings across this age range. First, we used eye movements to a landmark as our response to allow us to assess overt responses (e.g., a shift in gaze) even in prelinguistic infants. Second, we minimized demands on other cognitive systems in the task by measuring participants’ mental transformation of a visible object—rather than their memory for a previously seen object. This contrasts with previous studies that relied on participants’ overall visual preference scores (Lauer et al. 2015; Möhring and Frick 2013; Moore and Johnson 2008; Quinn and Liben 2014; Slone et al. 2018) or anticipatory looking in the absence of a forced choice (Pedrett et al. 2020, 2023).

Third, we structured our task to provide training on the response before we began the test phase, thus providing all participants with “instructions”. Importantly, this training was appropriate for even our youngest children. In our task, the children were first shown an initial demonstration phase containing an image of a giraffe and were taught to look at one location if the giraffe was facing right and at another location if the giraffe was facing left. Next, the children were presented with a practice phase to confirm that they understood the task. Finally, during the test phase, the children were shown multiple blocks containing individual trials in which the giraffe was shown at different orientations, requiring them to mentally rotate the image to determine where they should direct their gaze. During these test trials, we included an audiovisual reward when eye movements were made to the correct location to remind children of the goal (i.e., to look at the correct house) and to keep them engaged in the task. Finally, we presented the children with a large number of trials, allowing us to assess their responses to several different degrees of rotation. To tailor the session to each child’s mental rotation ability, we used a staircase procedure, in which the task became more difficult as the children successfully fixated on the correct location at a given angle of rotation and easier when they failed at a given angle.

## 2. Materials and Methods

### 2.1. Participants

All procedures were reviewed and approved by the Institutional Review Boards at Cornell University and UC Davis. Our final sample consisted of 41 children tested between 12 December 2019 and 3 December 2020; there were sixteen 12-month-old (M = 12.10 months, SD = 0.27 months, 10 girls), sixteen 24-month-old (M = 24.10 months, SD = 0.22 months, 6 girls), and nine 36-month-old children (M = 36.10 months, SD = 0.25 months, 6 girls). A total of 24 children were tested at UC Davis and 17 children were tested at Cornell University. The data collected were intended to be the first time point of a longitudinal study, but the study was discontinued due to the COVID-19 pandemic, yielding our current sample size. We tested an additional 15 children who were excluded from the final sample for the following reasons: 4 due to an inability to achieve an acceptable calibration, 3 due to experimenter error, 2 that became too fussy to advance to the experimental trials, 2 because of equipment malfunction, and 4 children demonstrated a lack of engagement with or understanding of the task (i.e., they not only failed the first experimental block but they also failed the subsequent probe trials) (see Section 2.4).

All parents of the children in the final sample reported educational attainment for the primary caregiver. Of the primary caregivers of the participants tested at Cornell University, 1 had less than a high school education, 2 completed some college, 3 earned a 2-year degree, 5 earned a 4-year degree, and 6 earned a graduate or professional degree. Of the primary caregivers of the participants tested at UC Davis, 1 had less than a high school education, 1 earned a high school diploma, 5 completed some college, 1 earned a 2-year degree, 9 earned a 4-year degree, and 7 earned a graduate or professional degree. Thus, the education levels of the primary caregivers were similar at the two sites. The parents also reported race and ethnicity information of their children who were included in our final sample. Of the children tested at Cornell University, 16 were White (1 of whom was also Hispanic) and 1 was Asian or Asian American. Of the children tested at UC Davis, 12 were White (3 of whom were also Hispanic), 2 were Asian or Asian American, 7 were more than one race (2 of whom were also Hispanic), and 3 did not report their race (all Hispanic). Thus, the UC Davis sample was somewhat more racially and ethnically diverse than the Cornell University sample.

### 2.2. Stimuli

**Figure 1:**
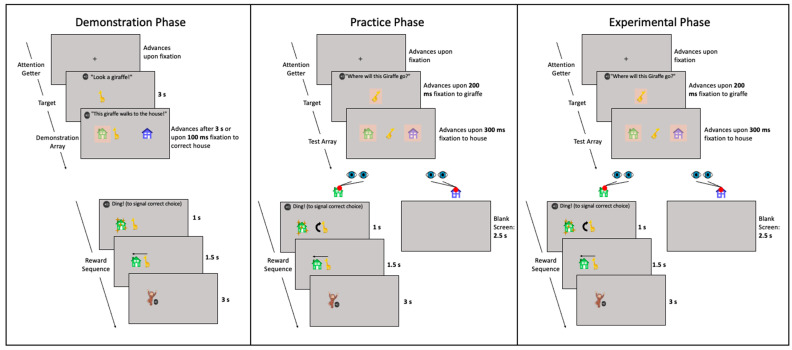
Schematic illustration of the experimental sequence and AOIs for the initial target (giraffe) and test array (houses). Note that the test array AOIs indexed anticipatory looking to the houses during the forced-choice aspect of the task.

The experimental visual stimuli consisted of cartoon images of a yellow giraffe that faced left or right, a blue house, and a green house (see Figure 1). The giraffe was 6.5 cm *×* 4 cm (6.20° × 3.82° at a viewing distance of 60 cm) and each house was 6 cm × 6 cm (5.72° by 5.72°). Each house was equidistant from the center of the screen, with a center-to-center distance of 12.75 cm (12.13°). The blue house was always presented on the right side of the screen and the green house was presented on the left side of the screen. A custom script was developed using the OpenCV (Bradski 2000) and matplotlib (Hunter 2007) Python libraries to generate clockwise and counterclockwise rotations of both the giraffe facing the left and the giraffe facing the right in 15° increments. All stimuli can be viewed in a demonstration video of the task that can be found on OSF (https://osf.io/ew3ug/). 

The experimental auditory stimuli were recorded phrases of a female voice directing the children’s attention to the stimuli. “Look, a giraffe!” and “This giraffe walks to the house” played during the demonstration phase when the giraffe first appeared on the screen and as the giraffe approached the house, respectively. During the practice and experimental phases, “Where will this giraffe go?” or “Where will this one go?” played when both houses appeared on the screen during the test display. 

In addition to these experimental stimuli, we used a center fixation cross that grew to 4.5 cm × 4.5 cm (4.30° × 4.30°) and shrank to 3 cm × 3 cm (2.86° by 2.86°). There were also several reward stimuli, each consisting of 3 s animated sequences in which a single cartoon character (i.e., a duck, Agnes from *Despicable Me*, Blue from Blue’s Clues, Brobee from Yo Gabba Gabba, Cookie Monster and Elmo from Sesame Street, Kermit from the Muppets, Curious George, Mickey Mouse, Nemo from *Finding Nemo*, Tigger, or Toad from the Super Mario Game Series) moved by bouncing up and down, oscillating back and forth, rotating, etc. Each reward animation was accompanied by audio segments of those specific characters and movement sequences for each character were generated in KeyNote. Sample videos of each reward sequence can be found on OSF. 

The stimulus used for calibration and validation was a swirling circle that grew to 5 cm (4.77°) in diameter and shrank to 1 cm (0.95°) in diameter. The specific stimuli used during calibration and validation have been shown to yield a high degree of accuracy and precision in infant eye-tracking research (Schlegelmilch and Wertz 2019). 

### 2.3. Apparatus

Eye-tracking data were recorded using an EyeLink 1000 Plus Eye tracker (SR Research, Ottawa, NO, Canada), using a 16 mm lens and 890 nm infrared illuminator, that recorded at a rate of 500 Hz (*n* = 8 at Cornell University, *n* = 18 at UC Davis) or 1000 Hz (*n* = 9 at Cornell University, *n* = 6 at UC Davis). The fact that the children were tested at different sampling rates had little impact on our results (see Discussion). The eye tracker was controlled by a Dell Laptop (Intel® CoreTM i7-7600U CPU @ 2.80 GHz 2.90 GHz). Experimental stimuli were presented on an ASUS vg248 24 in stimulus presentation monitor (1920 by 1080 resolution), which was controlled by an Ultra Performance Display PC (Intel® CoreTM i5-8600K CPU @ 3.60 GHz 3.60 GHz). The eye tracker and monitor were mounted on a hydraulic arm allowing the experimenter to flexibly adjust the position of the monitor and eye-tracking equipment based on the position of the child. Video recordings of participants were collected using an Anivia W8 1080p webcam (Lens: 3.6 mm, Power: DC 5 V) that was positioned immediately above the stimulus presentation monitor and captured the child’s face and upper body to monitor and record their overall movement and attention during the task.

### 2.4. Procedure

This eye-tracking task was conducted at the beginning of a session that involved multiple tasks (e.g., play tasks with puzzles, touch screen tasks). The data from these other tasks will not be reported here. The eye-tracking session occurred in a small room. The children were seated in a highchair at 12 months and a car seat at 24 and 36 months, and the parents sat behind their child (or with their child if the child became fussy in the car seat or highchair). The parents wore a pair of felt-covered glasses to help them refrain from looking at the stimuli.

Once the children were seated, the eye tracker and stimulus presentation monitor were adjusted (using the hydraulic arm) to position it approximately 60 cm from the child. The experimenters who were controlling the session sat behind a black curtain to hide them from the child’s view. Immediately prior to the eye-tracking session, a bullseye sticker was placed on the children’s forehead. The stickers—supplied by SR Research—provided a landmark for the eye-tracking system to localize the position of the children’s eyes in space.

Each session began with a 5-point calibration sequence in which a swirling shape was presented in the center, top center, middle left, bottom center, and middle right of the screen. The swirling shape expanded (5 cm × 5 cm, or 4.77° by 4.77°) and shrank (1 cm × 1 cm, or 0.95 by 0.95°) at each individual point (Schlegelmilch and Wertz 2019). When these stimuli were presented, the experimenter monitored the child’s gaze displayed on the eye-tracking laptop and manually accepted a fixation on the calibration stimulus by pressing a computer key once they judged that the child was attending to the location of that stimulus (as indicated by a letter superimposed over the location of the calibration stimulus). This key press triggered the shape to disappear and reappear at the next location in the sequence. Immediately after this process had been completed at all 5 locations, the experimenter pressed a button to manually advance the eye tracker to the validation procedure. During validation, the calibration stimuli were again presented in the same five locations, and the experimenter again pressed a computer key to manually accept the fixation once the child fixated each point. The children’s point of gaze (POG) was recorded for each of the five locations during validation, and accuracy was assessed by (1) visually inspecting the correspondence between the validation points and the calibration locations after validation was completed and (2) evaluating the calibration/validation quality codes generated by the EyeLink software. The EyeLink software automatically generated standardized codes to indicate whether the quality was poor, fair, or good based on the horizontal and vertical degrees of deviation observed during validation. The calibration process was repeated if the outcome was poor, fair, or if the experimenter judged upon visual inspection of the points that the child needed to be recalibrated. This process was repeated until a satisfactory calibration was achieved, or until it was clear that an adequate calibration was not achievable (recall that we tested 4 children whose data were discarded because adequate calibration could not be obtained).

The experimental procedure began immediately after the calibration/validation sequence was completed. The experimental procedure consisted of three phases: a demonstration phase, a practice phase, and an experimental phase. The goal of the demonstration phase was to introduce the children to the task in general. The practice phase was presented to confirm that the children understood the matching aspect of the task. During the experimental phase, the angle of rotation was manipulated using a staircase procedure, allowing us to identify each child’s maximum angle of rotation. Each phase consisted of multiple individual trials with a similar sequence (see Figure 1).

First, a center fixation cross preceded each trial. The children were required to look at the fixation cross to initiate the beginning of each trial. That is, the experimental software automatically presented the target stimulus after the children’s gaze fell within the trigger area of interest (AOI) surrounding the center fixation cross (5.55 cm × 5.55 cm, or 5.30° by 5.30°, at a viewing distance of 60 cm). Once the children looked at the fixation cross, a cartoon image of a giraffe was presented in the center of the screen as an initial target. Finally, two images of cartoon houses appeared to the left and right of the giraffe during the test array, and the giraffe (if upright) would be facing one of the two houses. The phases differed based on the timing of these events and what was required to progress to the next trial. Figure 1 displays a schematic illustration of the different phases of the task.

#### 2.4.1. Demonstration Phase

The demonstration phase consisted of three pairs of demonstration trials in which the children were presented with an upright cartoon giraffe (i.e., at 0° rotation). On the first of these pairs, the giraffe was presented near its “correct” house, and on each successive pair of trials the initial position of the giraffe moved closer to the center of the screen and farther away from the correct house (i.e., the house it was facing). The giraffe was presented in the center of the screen for the last pair of trials of the demonstration phase. This served to demonstrate to the children in a transparent way that the giraffe facing left was associated with the house on the left and the giraffe facing right was associated with the house on the right. Each pair consisted of a single trial in which the giraffe faced the house on the left and a single trial in which the giraffe faced the house on the right. 

When the giraffe (i.e., target) was presented, the children would hear “Look a giraffe!” After 3 s, the two houses appeared, one on the left and one on the right side of the screen. Each house was surrounded by a trigger AOI (9.72 cm × 9.72 cm, or 9.26° by 9.26°, at a viewing distance of 60 cm) that the eye tracker used to detect a fixation on one or the other house. To teach the children to fixate the correct house (i.e., the one the giraffe was facing), during each demonstration trial, once a child had fixated that house for 100 ms, the following sequence occurred: (1) a bell sound was played and the house that the giraffe was not facing simultaneously disappeared, (2) the correct house shimmered for 1 s, (3) a voice said “This giraffe walks to the house” and (in the second and third demonstration pairs) the giraffe approached the house, (4) the giraffe and house oscillated for 1.5 s, and (5) a 3 s reward sequence in which an animated character moved on the screen and made fun sounds was presented in the location of the correct house. If 3 s elapsed without the child fixating on the correct house, only steps 3 through 5 were presented to demonstrate which house the giraffe should walk toward.

#### 2.4.2. Practice Phase

The practice phase immediately followed the demonstration phase. The sequence and timing of these trials was similar to the demonstration trials except that (1) the houses were not presented until the children fixated the giraffe, (2) the children were required to fixate one of the two houses for 300 ms for the sequence to continue, and (3) fixations the correct house resulted in the reward sequence, but fixations to the incorrect house resulted in a blank screen. 

Each practice trial began with the giraffe (i.e., target) displayed in the center of the screen in an upright orientation (i.e., at 0° rotation) as the children heard “Where will this giraffe go?” or “Where will this one go?” Once the children fixated the AOI surrounding the giraffe (6.5 cm × 4 cm, or 6.20° × 3.82°, at a viewing distance of 60 cm) for 200 ms, the giraffe remained on the screen and the two houses appeared. This test array remained until the child fixated either house for 300 ms. If they first fixated the correct house, the following sequence occurred: (1) a bell sound was heard and the incorrect house disappeared, (2) a 2.5 s sequence was automatically presented in which the matching house shimmered, the giraffe approached the matching house, and the giraffe and house oscillated, and (3) a 3 s reward stimulus was then presented on the side of the screen in which the matching house was previously located. If the children first fixated the incorrect house, they saw a blank screen for 2.5 s. Note that the duration of the blank screen matched the timing of the animated sequence the children were shown when they executed a correct fixation (e.g., the sequence in which the house shimmered, the giraffe approached the house, and both the house and giraffe oscillated) and that the children received no 3 s reward stimulus in incorrect trials. 

The giraffe always faced the house on the left on the first practice trial. The children were required to pass a left-facing trial before moving on to a practice trial in which the giraffe faced right. For each face direction, the trials would repeat until the children directed their first fixation toward the correct house. That is, the children had to fixate the location of the correct house in one practice trial in which the giraffe was facing the house on the left and one practice trial in which the giraffe was facing the house on the right before advancing to the experimental phase. All the children included in the final sample passed the initial practice trials.

#### 2.4.3. Experimental Phase

During the experimental phase, the children were presented with up to 12 blocks, each containing four trials. On all the trials in a block, the giraffe was presented at a single rotational angle (e.g., 15°, 30°, 45°, 60°, 75°, 90°, 105°, 120°, 135°, 150°, 165°, 180°). The blocks presented to a child depended on their performance. All trials had the same sequence that differed from the practice trials in that the giraffe was initially presented in a clockwise rotation facing the left or right, as the children heard “Where will this giraffe go?” or “Where will this one go?” The face direction and rotation direction were randomly predetermined for each trial with the constraint that within each block the children saw one trial for each combination of rotation direction (clockwise vs. counterclockwise) and side (left facing vs. right facing). That is, in a given block, a child would see a clockwise rotated giraffe facing left, a counterclockwise rotated giraffe facing left, a clockwise rotated giraffe facing right, and a counterclockwise rotated giraffe facing right. The face and rotation direction order were the same for all participants but, as previously described, were randomly predetermined for each block.

The sequence within the trial was the same as in the practice trials: (1) Once the children fixated the giraffe for 200 ms, two houses appeared. (2) The giraffe and houses remained on the screen until the children fixated one of the two houses for 300 ms. (3) Fixations to the correct house resulted in the 2.5 s animated sequence (i.e., the matching house shimmered, the giraffe approached the matching house, and the giraffe and matching house oscillated) followed by a 3 s animated reward stimulus, whereas fixations to the incorrect house resulted in a 2.5 s presentation of a blank screen and no reward stimulus. Rewards were included to maintain the children’s interest in the task and motivate them to execute eye movements to the correct landmark based on their ability to mentally rotate the giraffe.

**Figure 2:**
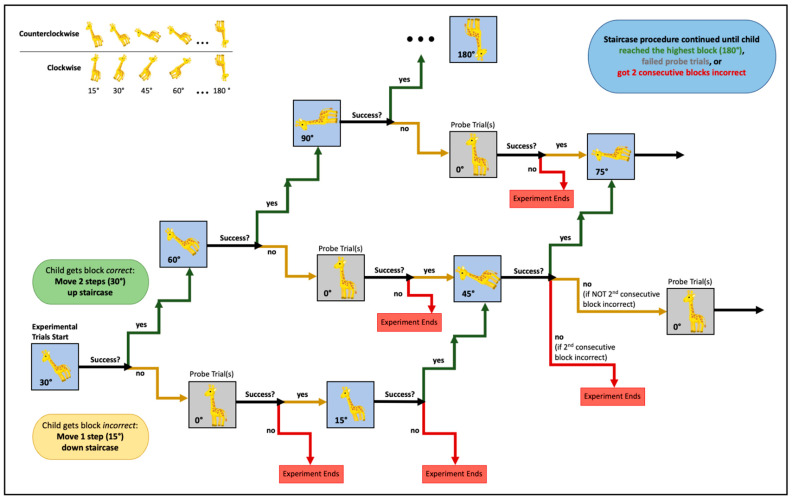
Schematic illustration of the staircase procedure.

The experimental phase was adaptive Ind progressed in a two-up, one-down staircase that incremented in 15° steps (see Figure 2). Success on a block was defined as making an eye gaze to the correct house in 3 of the 4 trials. For all children, the giraffe was rotated 30° in the first block for all four trials (once facing left rotated counterclockwise, once facing left rotated clockwise, once facing right rotated counterclockwise, and once facing right rotated clockwise). If the children looked at the correct house in 3 or 4 of the 30° trials, they progressed up the staircase two steps, or 30°, and next received a block of 4 trials in which the giraffe was rotated 60° in all 4 trials. 

If a child failed to look first at the correct house in at least 3 of the 4 trials in a block, it was determined that they failed. After failing a block, the children’s attention and engagement was immediately probed on one or more 0° trials to ensure that their failure truly reflected an inability to mentally rotate the object at that specific angle of rotation. If the children succeeded on the first 0° probe trial, they progressed down the staircase, but if they failed the first probe trial, they were presented with two more 0° probe trials (one to the left and one to the right). If they failed the first probe trial and failed on either of the two subsequent probe trials, the session ended. If they passed both probe trials, the task continued, and they progressed down the staircase.

Thus, the children progressed down the staircase only if they succeeded on the probe trials, and we were therefore confident that they were engaged and understood the game. The children who passed the probe trials immediately following any failed experimental block would be presented with a block of 4 trials that was one-step down from the set of trials presented in the previous experimental block. For example, if the children failed the 30° block, but then passed the probe trials, they would then be shown a block of 4 trials in which the giraffe was rotated 15°. To pass this block, the children would have to succeed on 3 of the 4 trials in that block. The staircase sequence continued until the children reached the highest possible angle of rotation (180°), failed the probe trial sequence, or failed two consecutive blocks despite passing the probe trials.

#### 2.4.4. Data Processing and Analytic Approach

The EyeLink software automatically parsed the eye-tracking data stream containing X and Y coordinates for the children’s point of gaze (POG) into fixations using the default Fast/Online algorithm. This algorithm parsed the raw data stream into saccades and fixations by imposing velocity and acceleration thresholds (velocity threshold = 35 deg/s, acceleration threshold = 8000 deg/s) across a sliding window while the session was being recorded. Saccades were automatically identified when both the velocity and acceleration thresholds were exceeded, and fixation labels were imposed for the duration of time in between saccades. Static areas of interest (AOIs) were defined in Data Viewer to label all fixations that occurred to the giraffe and house during the eye-tracking session and trial labels were generated to denote whether specific trials occurred during the demonstration phase, practice phase, or experimental phase. Response selection labels were imposed in Data Viewer to automatically assign a label indicating whether the location of the children’s first fixation was to the house on the left or right during the experimental phase. These data were then exported with labels indicating trial number, block number, videos and images presented, stimulus information about the orientation, face direction, and degree of rotation for the cartoon giraffe, and whether the children selected the house on the left or the house on the right in each trial. 

Data cleaning and statistical analyses were conducted in R version 4.1.1 (R Core Team 2013). Packages from the tidyverse were used to clean, manipulate, and visualize the data (Wickham et al. 2019), and statistical analyses were conducted using the ggsurvfit package (Sjoberg et al. 2023). Demographics data were imported, cleaned, and merged with the eye-tracking data in R to conduct our analyses. All scripts and data used for statistical analysis are available on OSF (https://osf.io/ew3ug/).

**Figure 3:**
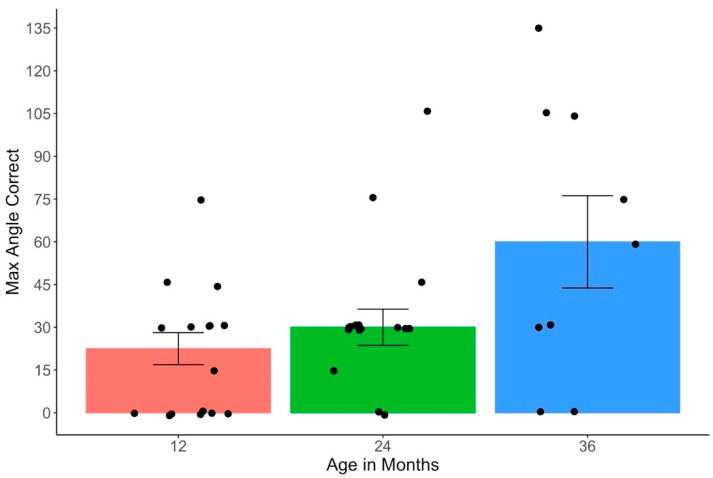
Children’s max angle correct for each age group. The height of the bars represents medians for each age group and the individual dots represent participant-level scores. The y-axis represents the max angle correct and the x-axis represents age group. Error bars indicate standard errors.

To ensure that only the children who understood the task were included in the analyses, we excluded the children who failed both the initial block of trials at 30° rotation and the probe trials that were presented after they failed that block. These criteria are stringent but ensure that all the children who were included in the final sample were engaged during the experimental phase, had learned the task structure, and were able to execute an eye movement to the landmark. Four children were excluded for failing to meet these criteria (see Section 2.2 and Section 2.4). Several measures were then derived to index the children’s performance in the staircase procedure. First, each child’s max angle was calculated by identifying the highest angle of rotation that they passed, or the block containing the highest angle of rotation that a given child was at least 75% correct. As can be seen in Figure 3, our data were skewed toward lower angles of rotation, with many children at each age achieving low max angles of rotation and a few children in each age group performing better than the others in each age group. Therefore, we calculated the median max angle correct to visualize the children’s performance in the task in each age group. Next, we used each child’s max angle of rotation to calculate a survival score for each child, defined as the angle of rotation one step (i.e., 15°) above the highest angle at which the children were successful in the staircase procedure. For example, if 45° was the highest angle at which a child succeeded, then their survival score was 60°. Thus, the survival score reflects the first angle of rotation that the children failed to pass.

The children’s survival scores were analyzed using proportional hazard regression analysis. Proportional hazard regression analysis is typically used to analyze health data and cumulative risk of disease progression. However, this approach has also been used in developmental research to index changes in behavior over time (Yanaoka et al. 2022). This approach is well-suited to investigating the effect of specific variables on the timing in which a specific event occurs (Breslow 1975). In the context of the present study, proportional hazard regression analysis was used to examine the effect of the increasing angle of rotation on the children’s accuracy. When predictors are included, the analysis yields a parameter estimate and p-value indicating whether a specific predictor significantly influenced the probability of survival. The parameter estimates generated from the proportional hazard regression analysis are on the logit scale but are typically exponentiated to generate hazard ratios (HR) in the same way that parameter estimates from logistic regression models can be transformed from the log scale into odds ratios (Szumilas 2010). Hazard ratios provide information about the rate of change in the probability of an event occurring as a function of a specific predictor. A hazard ratio > 1 indicates decreased likelihood of survival and a hazard ratio < 1 indicates increased likelihood of survival. Proportional hazard regression analyses are typically visualized using Kaplan–Meier curves (Kaplan and Meier 1958; Rich et al. 2010). These visualizations display the cumulative risk of an event occurring over time. Median survival estimates can also be derived from proportional hazard regression analysis to derive conclusions about changes in risk status over time. Median survival estimates represent the point at which 50 percent of participants survived. The outcome measure of these analyses is each child’s survival score (e.g., the angle immediately above their max angle of rotation). Including variables such as age, testing site, and child sex as predictors in the proportional hazard regression analysis yields parameter estimates and p-values indicating whether these variables significantly impacted the risk of failure in the staircase at specific angles of rotation.

To examine the children’s performance in the task, we fit a series of proportional hazard regression analyses with a survival score as the outcome measure. Our first model was fit to estimate the median survival time for the children as a group and thus included no predictors. The goal of this initial analysis was to derive a single estimate of the children’s performance overall. Our second model included age (continuous), testing site (categorical: Cornell University or UC Davis), sampling rate (categorical: 500 Hz or 1000 Hz), and child sex (categorical: male or female) as predictors, but we omitted testing site, sampling rate, and sex from subsequent models because controlling for these variables did not change the impact of age on our results. Our final model included age in years (continuous) as a predictor to examine whether child age impacted their probability of survival. Kaplan–Meier curves and median survival estimates were calculated for each age group to visualize how increases in the angle of rotation influenced the children’s probability of survival in the staircase.

## 3. Results

### 3.1. Task Engagement and Comprehension

To provide an overview of the children’s behavior in the task, we first assessed the total number of trials the children were presented with (including the six demonstration trials as well as practice trials, individual trials that were nested within experimental blocks, and probe trials). On average, the 12-month-old children were presented with 23.20 individual trials (SD = 5.89), the 24-month-old children were presented with 27.40 individual trials (SD = 8.09), and the 36-month-old children were presented with 31.70 individual trials (SD = 11.70). 

Next, we assessed the number of practice and probe trials the children received. Recall that the practice trials repeated until the children passed one practice trial in which the giraffe faced the house on the left and one in which the giraffe faced the house on the right. For this reason, the number of practice trials required to advance provides a measure of how quickly the children learned the goal of the task as well as their comprehension of the task. On average, the 12-month-old children required 4.44 practice trials (SD = 3.33), the 24-month-old children required 5.19 practice trials (SD = 4.62), and the 36-month-old children required 4.44 practice trials (SD = 2.74). Poisson regression analysis on the number of practice trials each child completed revealed no significant effect of age, ß = 0.02, SE = 0.09, z = 0.20, *p* = .84. Thus, at all three ages the children learned the task and displayed similar comprehension as measured by the number of trials presented during the practice phase.

Recall that probe trials were presented during the experimental phase following any four-trial block in which the children failed (i.e., were successful in fewer than three out of four trials in a block). Because the number of probe trials varied after each block (depending on the children’s performance), we report the number of blocks that were followed by one or more probe trials, or, in other words, how often the children’s engagement and comprehension of the task was evaluated after failing a block. On average, across the entire session, the 12-month-old children received one or more probe trials after 1.20 (SD = 0.41) blocks, the 24-month-old children received one or more probe trials after 1.47 (SD = 0.64) blocks, and the 36-month-old children received one or more probe trials after 1.56 (SD = 1.01) blocks. Poisson regression analysis on the number of times in which one or more probe trials was presented after a block revealed no significant effect of age, ß = 0.13, SE = 0.18, z = 0.76, *p* = .45.

### 3.2. Staircase Analysis

To analyze the children’s mental rotation performance in the staircase procedure, we first calculated the children’s median max successful angle of rotation for each age group (Figure 3). The median max angle of rotation was 22.5° at 12 months of age, 30° at 24 months of age, and 60° at 36 months of age (see Figure 3). As can be seen in Figure 3, 11 children’s max angle was 0°. In our procedure, to receive a max angle of 0°, a child would have to have failed the initial 30° block, succeeded on the 0° probe trial sequence, and then subsequently failed on the 15° block. Thus, these children learned the task, were successful at 0°, but were unable to succeed when the giraffe was rotated even 15°.

**Figure 4:**
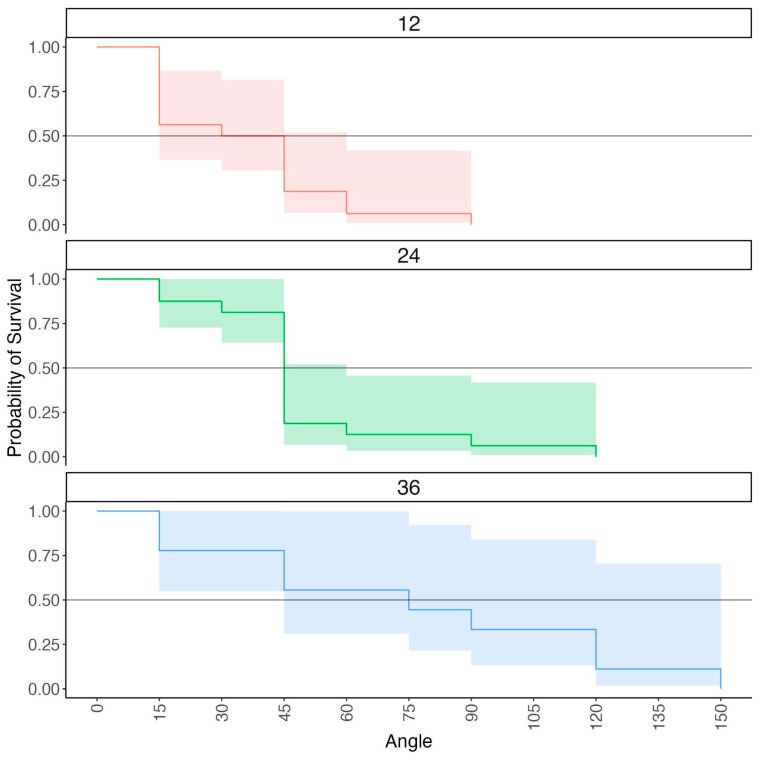
This Kaplan–Meier curve depicts the probability of children, separated by age group, surviving through the staircase as angle of rotation increases. The x-axis indicates angle of rotation, and the y-axis represents children’s probability of survival. Shading around the lines represents 95 percent confidence intervals.

Our next analysis examined the children’s performance in our task using a survival analysis (see analytic approach). A proportional hazard regression analysis was conducted with the children’s survival scores as the outcome measure (i.e., the angle 15° above each child’s max successful angle). Our first analysis collapsed across age (i.e., age was omitted in the model). Examination of the children’s survival time revealed that 68% of the children succeeded up to 30°, 95% *CI* [55%, 84%]; some of these children succeeded at higher angles, and for some, this was their max angle. A second proportional hazard regression analysis included age (continuous) as a predictor. For this analysis, age was dummy coded in years to derive hazard ratios for each age group in the present study. The hazard ratios revealed a significant effect of age on the children’s probability of success, *HR* = 0.56, 95% *CI* [0.37, 0.87], *p* = .01. Figure 4 displays the survival curves for each age group. This result indicates that a 12-month increase in age yielded a 44 percent increase in the probability of success in the staircase, meaning that, overall, older children were more likely to succeed across all angles of rotation than younger children. This analysis revealed that only 50% of the children at 12 months succeeded at 30° (95% *CI* [31%, 82%]), whereas 81% of the children at 24 months of age (95% *CI* [64%, 100%]) and 78% at 36 months of age (95% *CI* [55%, 100%]) succeeded at 30°.

### 3.3. Exploring Variation in Children’s Pathways to Achieving Their Max Angle

**Figure 5:**
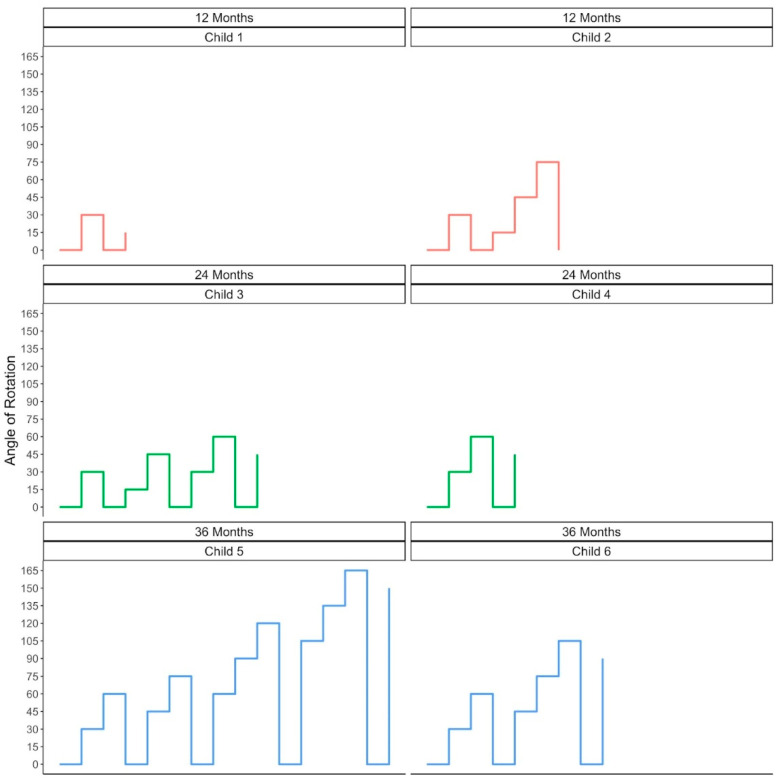
Examples of 6 randomly selected children’s progression through the staircase. Note that the progression includes zero probe trials when children failed a block, i.e., points in which the lines dropped to zero. The x-axis indicates time through the task (the leftmost point reflects the practice trials at 0°, followed by a block at 30°, and so on). The y-axis indicates angle of rotation. Each plot represents a single participant and individual lines indicate their progression through the staircase.

The survival analysis above provides evidence for age-related changes in the children’s progression through the staircase but does not allow us to examine the pathways that individual participants followed as they progressed through the staircase. To explore individual differences in our sample, we visualized the angles that the children were presented on each block as a measure of their progression through the staircase. We randomly selected a subset of participants from each age group to demonstrate differences in the pathways the children followed to achieve their max angle of rotation (see Figure 5). 

Several things are clear from Figure 5. As was evident in Figure 3, Figure 5 shows that some children reached a higher max angle than other children, even within the same age group. Second, the children did not tend to move steadily up the staircase but instead often took two steps forward and one step back. Finally, the children took different pathways to reach the same max angle of rotation. This is clear when comparing the two 24-month-old children in Figure 5 (Child 3 and Child 4). Both children achieved the same max angle (60°), but Child 4 did it by steadily moving up the staircase, whereas Child 3 progressed both up and down the staircase. These variations in the pathways the children followed as they progressed through the staircase may be indicative of individual differences in children’s mental rotation. 

## 4. Discussion

Mental rotation is a critically important spatial cognitive skill that predicts educational achievement in children (Casey et al. 2015; Geer et al. 2019; Gilligan et al. 2017; van Tetering et al. 2019) and adults (Hegarty and Kozhevnikov 1999; Johnson and Bouchard 2005; Shepard and Metzler 1971). Fewer studies have linked mental rotation during the first years of life to long-term academic and career outcomes (Lauer and Lourenco 2016). A key barrier to addressing this disparity in the literature is the lack of a single task for indexing mental rotation from infancy through early childhood. Previous studies have assessed mental rotation in infants and other studies have done so in young children, but to our knowledge the present study is the first to examine this key spatial cognitive ability in children from infancy into the preschool period using a single task. We provide evidence that a novel adaptive eye-tracking task can assess mental rotation in children between 12 and 36 months. Such a tool is critical for evaluating not only the development of mental rotation in the first years but also the impact of interventions on spatial skills and math achievement across childhood. 

One challenge in assessing mental rotation from infancy to preschool age is finding a task that is both engaging and developmentally appropriate across this age span. Typically, mental rotation in infancy is assessed with looking-time measures (for review, see Moore and Johnson 2020), whereas studies in preschoolers often use choice tasks, presenting children with a set of stimuli and asking them to choose images that are rotations of the same object versus images that are mirror images (Estes 1998; Kail 1986; Marmor 1975; Quaiser-Pohl et al. 2010). We developed a procedure that uses children’s looking behaviors to indicate a choice. Our participants had to execute a shift in eye gaze to one of two options, reflecting their mental rotation of a central target (the giraffe) to decide which option was correct. Although we measured the children’s looking behavior, we did so in a way that mimics the mental rotation tasks used with older children that require them to select between response options (e.g., identical or mirror or which options match a target). Thus, we used a measure that was appropriate for even the youngest children while simultaneously requiring the children to produce a choice, allowing us to sample children’s’ mental rotation abilities across a wide age range (e.g., from 12 to 36 months of age). 

Several additional design features were incorporated into the procedure to ensure that the task was developmentally appropriate across this age range. To ensure comprehension of the task, the participants were first trained on the task using a demonstration phase followed by a series of practice trials. We required that the children pass the practice trials before advancing to the experimental phase of the study, thus demonstrating they understood the general task of making a choice by making an eye movement. This procedure was successful. Only six of the children we tested failed to demonstrate comprehension in this task as assessed by the practice trials or by the first experimental block and following probe trials. Moreover, our task appeared to be similarly difficult to learn across the age range we tested. At each age, participants completed on average four to five practice trials, and there were no significant differences across our age groups. The lack of age differences in the number of practice trials children received before advancing to the experimental phase suggests that, despite the two-year age range, participants at all ages grasped the directionality of the giraffe and learned to match the giraffe to the correct house. To ensure engagement and comprehension of the task, reward stimuli were included when participants passed a block, and 0° probe trials were included whenever children failed a block. Rewards were included to motivate the children to execute eye movements to the correct landmark and increase engagement in the task, whereas probe trials were included to verify that the children’s failure at a given angle reflected an inability to mentally rotate the object at the angle of rotation presented (rather than fatigue or disinterest in the task). Taken together, these design features reduced the likelihood that any observed differences across age groups were due to variation in comprehension or engagement.

In addition, the staircase procedure offers several advantages for indexing mental rotation across our wide age range of young children. For one, the inclusion of gaze-contingent eye tracking allowed us to adaptively sample the children’s response to angles of rotation based on their performance. Because the children were required to succeed at one angle of rotation prior to progressing to a higher angle of rotation, the participants only viewed angles of rotation within a narrow window defined by their success at specific angles. As a result, the children were shown many trials without requiring the presentation of many angles of rotation beyond their capability, minimizing the risk of a floor effect reported in several studies of mental rotation with young children (e.g., Estes 1998; Frick et al. 2013; Hawes et al. 2015). At the same time, children with more advanced abilities can progress quickly through the staircase. Another aspect of the staircase procedure that makes it ideal for indexing mental rotation across this age range is the ability to probe a broader range of angles than is possible in non-adaptive procedures. Typically, studies of mental rotation in children have sampled only specific angles that differ in 45° increments (Frick et al. 2013). In our procedure, children were tested on 30° or 15° increments based on their performance. This increment provided us with a higher degree of precision in estimating children’s mental rotation than is possible in other procedures. Moreover, our task allowed us to present blocks at a specific angle of rotation that each contained multiple individual trials, providing us with a large number of observations for each participant.

One question these findings raise is whether the staircase procedure used in the present study provides a valid measure of mental rotation across this age range. Importantly, several findings in the present study align with previous research on mental rotation. As has been found in other studies, particularly studies with older children (Ebersbach and Nawroth 2018; Frick et al. 2013; Krüger et al. 2014; Marmor 1975, 1977) and adults (Cooper and Shepard 1973; Vandenberg and Kuse 1978), performance on our task varied with the angle of rotation. There is some evidence of angular disparity effects on mental rotation in infants (Gerhard and Schwarzer 2018), but fewer studies have examined this question in infants. In each of these studies, as in our study, participants were most successful on smaller angles of rotation and less successful as the angle of rotation increased.

In addition, as has been observed in previous studies, the older children in our study were more successful overall and at higher degrees of rotation than were the younger children. This pattern of accuracy improving with age is consistent with several studies that have shown that preschoolers and older children display greater accuracy, faster response times, and more consistent above-chance performance in mental rotation tasks than younger children (Frick et al. 2013; Pedrett et al. 2023; Wimmer et al. 2017).

Recall that we used audiovisual rewards during the experimental or test trials to maintain the children’s engagement and to remind them of the goal of the task. The inclusion of these rewards was a key feature that we believe is important to the success of the task. One might be concerned that the inclusion of rewards could have introduced learning effects. Specifically, by rewarding the children for their correct response, it might appear that the children learned to mentally rotate across the experimental session. Indeed, this is why we used rewards during the demonstration and training—to help the children learn that the goal of this task was to match the giraffe to the correct house. However, it is unlikely that the rewards helped the children learn to mentally rotate across trials in the experimental phase. Success in this task requires that the children mentally rotate. If they performed at chance on the first block, they did not advance in the staircase. It is possible that they rapidly learned from their failure on the first block to success on the second block how to mentally rotate, or that mental rotation was relevant, but because progression required a high level of success, the children could not advance beyond this point if they were learning how to mentally rotate in the context of the task.

Our stimuli involved presenting a cartoon giraffe with a face that was oriented in a specific direction. Because gaze following is well-documented during infancy and early childhood (Gredebäck et al. 2018; Senju and Csibra 2008; Farroni et al. 2004; Brooks and Meltzoff 2005; Lee et al. 1998), it is important to determine whether the present results reflect the children’s ability to follow the gaze direction of the giraffe rather than their mental rotation. Recall that each block consisted of four trials in which clockwise or counterclockwise rotations were shown (see Figure 1). Thus, because the giraffe was sometimes facing up and sometimes facing down, its gaze direction varied on clockwise and counterclockwise rotations. Moreover, following the giraffe’s gaze to the house would have required the children to mentally rotate the giraffe in the appropriate direction to determine which house it would be facing if it were unrotated. That is, it is possible that the children were able to rely on the giraffe’s gaze direction to execute an eye movement to the correct landmark but doing so would still require them to mentally rotate the giraffe to align its gaze with the correct landmark. Therefore, it is unlikely that gaze following alone would enable children to succeed in the task.

It also must be pointed out that although we observed mental rotation in our youngest 12-month-old children, as a group they succeeded only at a relatively low degree of rotation (between 20° and 30°), which contrasts with other studies in which infants succeeded at much higher degrees of rotation (Gerhard and Schwarzer 2018). One explanation for this discrepancy is the tasks used to index mental rotation. Most studies that have demonstrated mental rotation in infants have used habituation or familiarization procedures in which infants are repeatedly presented with a single stimulus multiple times over several seconds (for review, see Moore and Johnson 2020). These tasks typically require between-subjects manipulations, which do not allow for precise estimates of the mental rotation abilities of individual children. One difference between habituation designs and our task is that in the present study, the participants were required to make in-the-moment decisions about how the object was rotated. Another difference is that we were able to manipulate the angle of rotation within-subject. These different procedures may tap different aspects of mental rotation in infants and young children.

Finally, in this study, we inadvertently used two different sampling rates when collecting data. It is possible that the 500 Hz sampling rate is less sensitive and as a result it may introduce more errors. In our sample, we observed that one child tested with a 1000 Hz sampling rate would have received slightly different final scores (one step lower) if they had been tested at 500 Hz. However, the different sampling rates were distributed randomly across our sample, with similar proportions of the children receiving the 500 Hz sampling rate at the youngest and oldest age groups, and approximately the same number of children receiving the 1000 and 500 Hz sampling rates at 24 months. Thus, the differences in performance across the ages were not due to the younger children having been assessed using a less sensitive test. Moreover, our saccade thresholds (used to identify fixations) were conservative and minimized the presence of short fixations in our data, which are the fixations most likely to be differentially classified when using different sampling rates. Finally, we included the sampling rate in our model, and it did not yield any significant effects, demonstrating that there was no systematic effect of the sampling rate on the children’s scores. Thus, although we advise using the highest sampling rate possible when adopting a procedure like that used here, we believe that the use of two sampling rates in our study did not significantly influence the outcome.

### Limitations

Although our task is a promising new tool for studying mental rotation in infancy and early childhood, there are some limitations to this study. First, despite the potential of this task for assessing the developmental trajectory of mental rotation and individual differences in this critically important spatial skill, we did not have a sufficient sample size for exploring these possibilities. For example, our task could provide a measure of children’s reaction time (i.e., latency to look at the correct look); reaction time has shown individual differences in adults’ mental rotation (Jansen et al. 2012; Krause et al. 2021) and has been used to reveal the developmental trajectory of mental rotation in children (Kail 1986; Perrucci et al. 2008). In addition, it may be possible to quantify the differences in individual trajectories through the staircase as illustrated in Figure 5. Those differences in individual trajectories may be reflective of individual differences in mental rotation abilities. Future research with larger sample sizes will be able to examine the effectiveness of such measures (e.g., reaction time and staircase trajectories) for uncovering both individual differences and developmental trajectories in this spatial ability.

A second limitation is that, as is common with experimental procedures assessing mental rotation, it remains to be established how performance in our task relates to children’s mental rotation in their natural environments. Children engage in mental rotation many times every day as they rotate a puzzle piece into a correct aperture, recognize a block from different viewpoints, or flexibly adjust their actions as they insert a shape into a shape sorter. Thus, in contrast to typical lab procedures, in children’s everyday lives they mentally rotate in service of their actions on objects. Thus, it is difficult to generate robust claims about the ecological validity of our task, along with most experimental procedures developed for this purpose. The fact that the children’s performance in our task displayed characteristics that have been previously reported in mental rotation studies (e.g., age-related change and angular disparity effects) lends support to the conclusion that our task is indexing and isolating mental rotation in the same way as other lab-based assessments. However, a larger issue is how children’s performance in such tasks is associated with how children implement mental rotation in the service of spatial activities. Future research will be necessary to explore how children’s mental rotation performance in this task relates to more naturalistic behavior.

## 5. Conclusions

In conclusion, the present study addresses a significant gap in our understanding of the development of spatial skills by reporting findings from a novel eye-tracking staircase procedure. Our findings suggest that this procedure—or tools like it—may be useful for bridging the gap in our understanding of mental rotation from infancy through early childhood and addressing disparities in the existing mental rotation literature. Isolating the development of spatial skills such as mental rotation from other cognitive processes across this age range is critically important for developing interventions aimed at improving spatial skills and improving disparities in mathematics and STEM achievements. The present study highlights a novel tool for characterizing the developmental trajectory of this fundamentally important spatial skill.

## Data Availability

The data and analysis scripts for the results reported in the present study are openly available through the Open Science Foundation at https://osf.io/ew3ug/.

## Note

1 Angular disparity effects can be examined between-subject in infant paradigms, but such manipulations are challenging to implement and we are only aware of one study that examined angular disparity effects in infants (Gerhard and Schwarzer 2018).
